# Effects of curcumin on retinal oxidative stress and inflammation in diabetes

**DOI:** 10.1186/1743-7075-4-8

**Published:** 2007-04-16

**Authors:** Renu A Kowluru, Mamta Kanwar

**Affiliations:** 1 Kresge Eye Institute, Wayne State University, Detroit, MI, USA

## Abstract

**Background:**

Oxidative stress and inflammation are implicated in the pathogenesis of retinopathy in diabetes. The aim of this study is to examine the effect of curcumin, a polyphenol with antioxidant and anti-inflammatory properties, on diabetes-induced oxidative stress and inflammation in the retina of rats.

**Methods:**

A group of streptozotocin-induced diabetic rats received powdered diet supplemented with 0.05% curcumin (w/w), and another group received diet without curcumin. The diets were initiated soon after induction of diabetes, and the rats were sacrificed 6 weeks after induction of diabetes. The retina was used to quantify oxidative stress and pro-inflammatory markers.

**Results:**

Antioxidant capacity and the levels of intracellular antioxidant, GSH (reduced form of glutathione) levels were decreased by about 30–35%, and oxidatively modified DNA (8-OHdG) and nitrotyrosine were increased by 60–70% in the retina of diabetic rats. The levels of interleukin-1β (IL-1β) and vascular endothelial growth factor (VEGF) were elevated by 30% and 110% respectively, and the nuclear transcription factor (NF-*k*B) was activated by 2 fold. Curcumin administration prevented diabetes-induced decrease in the antioxidant capacity, and increase in 8-OHdG and nitrotyrosine; however, it had only partial beneficial effect on retinal GSH. Curcumin also inhibited diabetes-induced elevation in the levels of IL-1β, VEGF and NF-*k*B. The effects of curcumin were achieved without amelioration of the severity of hyperglycemia.

**Conclusion:**

Thus, the beneficial effects of curcumin on the metabolic abnormalities postulated to be important in the development of diabetic retinopathy suggest that curcumin could have potential benefits in inhibiting the development of retinopathy in diabetic patients.

## Background

Diabetic retinopathy is the main cause of acquired blindness in working adults. Abnormalities in retinal metabolism, including elevated polyol pathway activity, increased nonenzymatic glycation and advanced glycation end products, oxidative stress, protein kinase C (PKC) activity [1-5], evidently contribute to the development of retinopathy, but the exact mechanism is still elusive. In diabetes the retina experiences increased oxidative stress [4,6-8], and reactive oxygen species (ROS) are considered as a causal link between elevated glucose and the metabolic abnormalities important in the development of diabetic complications [9]. However, the mechanism by which oxidative stress can contribute to the development of diabetic retinopathy remains to be elucidated.

Recent studies have compared the development of diabetic retinopathy to the low-level chronic inflammatory disease; the retinal capillaries become nonperfused and ischemic, and the number of platelet-fibrin thrombi increases in diabetes [10]. The levels of pro-inflammatory cytokines are increased in the retina and vitreous in diabetes [11,12]. We have shown that intravitreal injection of interleukin-1β (IL-1β) to the normal rats increases retinal capillary cell apoptosis and histopathology; and these IL-1β-induced changes in the retinal capillaries of normal rats are similar to those observed in diabetes [13]. Further, IL-1β is shown to increase the expression of vascular endothelial growth factor (VEGF) in retinal endothelial cells [14], and VEGF is implicated in the development of diabetic retinopathy [15].

**Figure 1:**
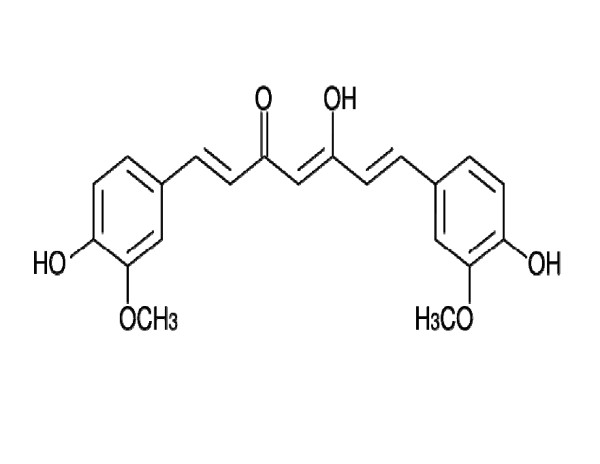
**Structure of curcumin**. 1,7-bis (4-hydroxy-3-methoxy-phenyl) hepta-1, 6-diene-3, 5-dione).

Curcumin, a bis-α, β-unsaturated β-diketone (Figure 1), possesses diverse antioxidant and anti-inflammatory properties [16,17]. It significantly decreases lipid peroxidation, increases intracellular antioxidant, GSH, regulates antioxidant enzymes, and scavenges hyperglycemia-induced ROS [18,19]. In addition, curcumin is shown to inhibit the pro-inflammatory transcriptional factor, NF-*k*B, and prevent up-regulation of VEGF mRNA and microvascular angiogenesis [20]. However, the beneficial effect of curcumin on diabetic retinopathy remains to be explored.

In the present study we have investigated the effect of administration of curcumin on oxidative stress and inflammatory markers in the retina of diabetes. The total antioxidant capacity, and the levels of GSH, oxidatively modified DNA (8-OHdG), nitrotyrosine, IL-1β, NF-*k*B and VEGF were quantified in the retina of diabetic rats that received diets supplemented with or without curcumin for 6 weeks, and for comparison, in the retina of age-matched normal control rats. The results presented show that curcumin administration for 6 weeks prevents diabetes-induced increase in retinal oxidative stress and inhibits the levels of pro-inflammatory markers.

## Methods

### Rats

Lewis rats (200 to 220 g, male) were made diabetic with streptozotocin (55 mg/kg body weight). Insulin was administered to diabetic rats to allow slow weight gain while maintaining hyperglycemia (blood glucose levels of 20–25 mM). Age-matched normal rats served as control. Diabetic rats were divided into 2 groups: the rats in group 1 received powdered diet (Purina 5001) without any supplementation, and group 2 received diet supplemented with curcumin (0.5 g/kg diet); these diets were initiated soon after establishment of diabetes (3–4 days after administration of streptozotocin). Each group had 8–12 rats, and the entire rat colony received fresh powdered diet weekly. The rats were weighed two times a week and their food consumption was measured once every week. After 6 weeks of diabetes the rats were euthanized by an overdose of pentobarbital, the eyes were removed, and retina was isolated and frozen immediately in liquid nitrogen for biochemical measurements. Treatment of the animals conformed to the National Institutes of Health Principles of Laboratory Animal Care, the Association for Research in Vision and Ophthalmology Resolution on the Use of Animals in Research, and the Institutional guidelines.

### Oxidative stress

The total antioxidant capacity of the retina was measured using a kit from Cayman Chemical (Ann Arbor, MI) according to the method recently used by us [21]. The assay is based on the ability of the sample to inhibit oxidation of 2,2'-Azino-di- [3-ethylbenzthiazoline sulfonate]^+ ^(ABTS) by metmyoglobin; the antioxidants in the sample cause the decrease in absorbance at 750 nm, and that represents the amount of ABTS^+ ^produced. Each sample was measured in duplicate.

The levels of 8-OHdG were quantified by competitive ELISA (Oxis Research Laboratories, OR) using 15–20 μg DNA purified from the retina that has been digested with DNAse, as described previously [22].

GSH was measured using the Glutathione Assay Kit from Cayman Chemical (Ann Arbor, MI) [22]. Fifteen to 25μg retinal protein was deproteinized using phosphoric acid, and the supernatant was used to measure the amount of 5-thio-2-nitrobenzoic acid.

Peroxynitrite is formed by the reaction between superoxide and nitric oxide, and nitrotyrosine is a measure of peroxynitrite. Nitrotyrosine levels were quantified by enzyme immunoassay using a Nitrotyrosine-EIA kit from Oxis Research, Portland, OR, as previously used by us [22]. The sensitivity of the assay was as low as 0.05 pmoles of nitrotyrosine.

### Inflammation

The amount of IL-1β was quantified in the retina by ELISA (R&D Systems, Minneapolis, MN) as previously described by us [12]. Unbound substances were removed by incubating the samples with rat polyclonal antibody in pre-coated microplates. The results are reported as pg of IL-1β/mg retina protein in each sample.

VEGF levels were quantified in the retina by an ELISA method using a kit from R&D Systems, MN. The standard solution or the samples were added in a 96-well plate that was pre-coated with a monoclonal antibody. The samples were incubated for 2 hours, and after washing the plate the samples were incubated with rat VEGF-conjugate. The assay was sensitive to the concentration of VEGF as low as 15 ng/ml.

The activation of the transcription factor that is under the control of IL-1β, NF-*k*B, was determined by ELISA that is based on the principle that only the active form of NF-*k*B in the sample binds to oligonucleotide containing NF-*k*B consensus site (5,-GGGACTTTCC-3'), which is immobilized on the microtiter plate. Eight-10 μg of retina protein was sufficient to provide reliable readings [22].

Protein expressions of IL-1β, VEGF and p65 subunit of NF-*k*B were determined by western blot using polyclonal antibodies against IL-1β, VEGF and p65 respectively. The membranes were developed using ECL-Plus western blotting reagents, and β-actin was used as a house-keeping protein.

### Statistical analysis

Data are reported as mean ± SD. The results were analyzed using one-way ANOVA followed by Fischer's test. Similar conclusions were reached by nonparametric Kruskal-Wallis, test followed by Mann-Whitney test for multiple group comparison.

## Results

### Oxidative stress

**Figure 2:**
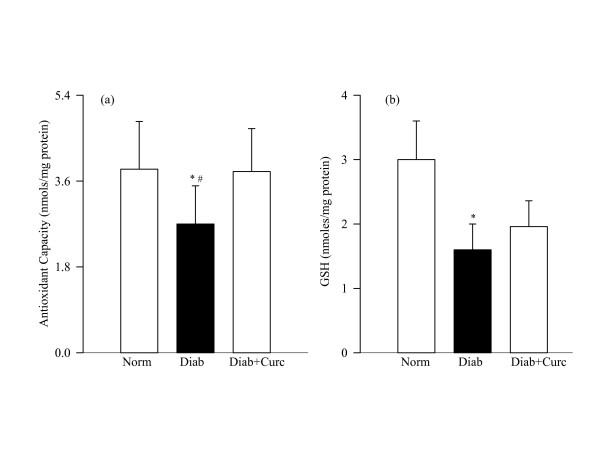
**Effect of curcumin on diabetes-induced oxidative stress in the retina**. (a) Total antioxidant capacity of the retina was quantified by measuring the ability of the retina to inhibit oxidation of ABTS by metmyoglobin using 5–10 μg retina protein. (b) Retinal GSH was estimated in the deproteinizing retinal homogenate using a kit from Cayman Chemical (MI). Each sample was measured in duplicate, and the values are represented as mean ± SD of 7–8 rats in diabetes group and 7–9 rats each on normal and diabetes + curcumin groups. Norm = normal; Diab = Diabetes; Diab + Curc = Diabetes + curcumin treated rats. *P < 0.05 compared to normal, ^#^P < compared to diabetes + curcumin.

**Figure 3:**
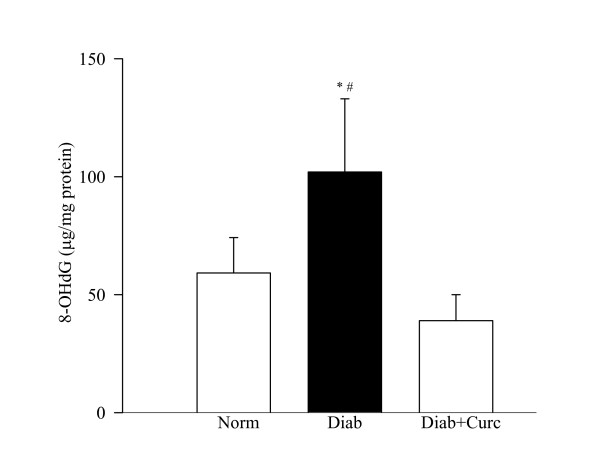
**Effect of curcumin on oxidatively modified DNA levels in the retina**. 8-OHdG levels were measured in the retina using an ELISA kit from Oxis Research. Values are represented as mean ± SD of 6–8 rats in each group. *P < 0.05 compared to normal, and ^#^P < 0.05 compared to diabetes + curcumin.

Oxidative stress, as determined by the overall antioxidant capacity, concentration of the intracellular antioxidant and the levels of oxidatively modified DNA remained elevated in the retina of rats diabetic for 6 weeks. The antioxidant capacity of the retina and GSH levels were decreased by about 30–35% (Figure 2), and 8-OHdG levels were elevated by over 70% (Figure 3) in diabetes compared to the age-matched normal control rats.

Administration of curcumin prevented diabetes-induced decrease in the total antioxidant capacity of the retina; the values obtained from normal control rats and in curcumin-treated diabetic rats were similar (Figure 2a). However, diabetes-induced decrease in retinal GSH was partially inhibited by curcumin administration; although GSH levels were lower in the retina of curcumin-treated diabetic rats compared to the normal control rats, these values were significantly higher compared to the diabetes group (P = 0.04; Figure 2b). In the same rats, curcumin administration had significant beneficial effect on oxidative modification of retinal DNA; 8-OHdG values in curcumin-treated diabetic group and normal control group were not different from each other (P = 0.32 compared to normal, Figure 3).

**Figure 4:**
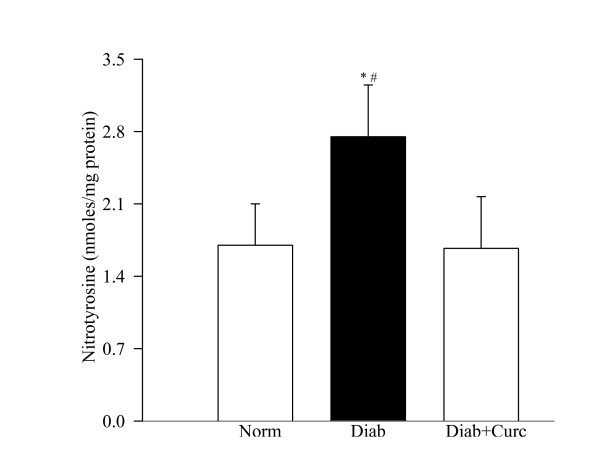
**Effect of curcumin on retinal nitrotyrosine levels in diabetes**. Nitrotyrosine-EIA kit was used to quantify nitrotyrosine in the retina. Each sample was measured in duplicate. The figure represents mean ± SD of 6 rats each in normal and diabetes + curcumin groups and 7 rats in diabetes group. *P < 0.05 compared to normal, ^#^P < compared to diabetes + curcumin.

Six weeks of diabetes increased nitrotyrosine levels in the retina by about 60% compared to the values obtained from normal control rats (Figure 4). Curcumin supplementation in diabetic rats prevented increase in retinal nitrotyrosine levels; the values in the curcumin-treated diabetic rats were significantly lower compared to diabetic rats without curcumin (P < 0.05, Figure 4).

### Inflammation

**Figure 5:**
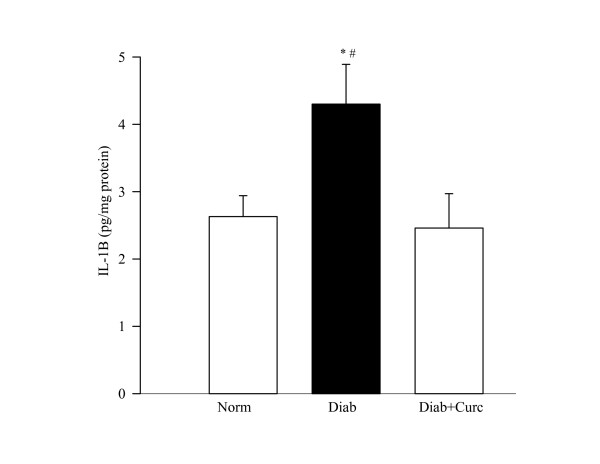
**Effect of curcumin on diabetes-induced increase in retinal IL-1β levels**. An ELISA kit from R&D Systems, Minneapolis, MN, was used to quantify IL-1β in the retina. Each sample was run in duplicate to ensure reproducibility of the data. IL-1β values (pg IL-1β/mg protein) are mean ± SD obtained from 8 rats each in diabetes and diabetes + curcumin groups and 6 rats in normal group. *P < 0.05 and ^#^P < 0.05 compared to normal and diabetes + curcumin groups respectively.

**Figure 6:**
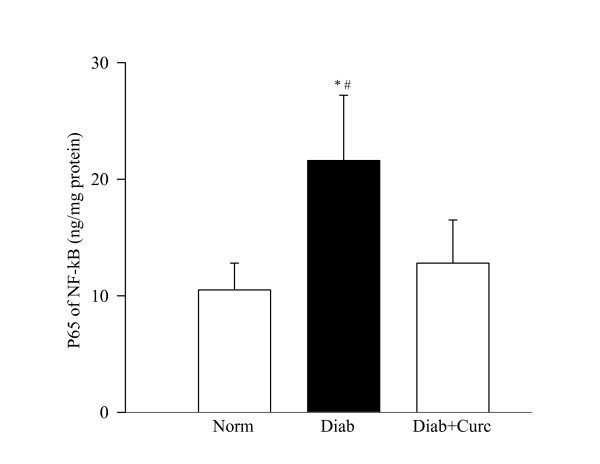
**Effect of curcumin on NF-*k*B activation**. NF-*k*B activation was determined in the retinal homogenate by ELISA method (kit from Active Motif.) using antibody specific for p65 subunit of NF-*k*B. Secondary antibody conjugated to horseradish peroxidase was used to quantify the activated form spectrophotometrically. The figure represents mean ± SD of 5–6 rats each in of the 3 groups. *P < 0.05 compared with normal or diabetes + curcumin groups.

The concentration of the inflammatory cytokine IL-1β and its protein expression were elevated by 30% in the retina of diabetic rats (Figure 5). In the same retina, the transcription factor that is under the control of IL-1β, NF-*k*B, was also activated by about 2 fold (Figure 6). Administration of curcumin inhibited diabetes-induced increase in inflammatory markers in the retina; the levels of IL-1β were significantly decreased in diabetic rats receiving curcumin (P < 0.05), and in the same retina the activation of NF-*k*B was also diminished.

**Figure 7:**
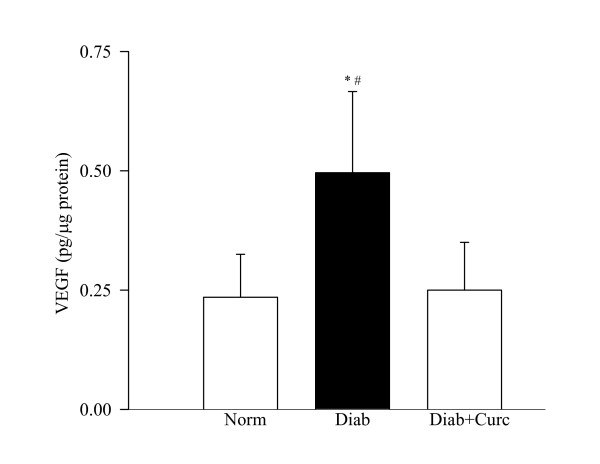
**VEGF levels in curcumin-treated rats**. VEGF concentrations were measured in the retina of rats in normal, diabetes and diabetes + curcumin groups using an ELISA kit from R&D Systems. Results are presented as mean ± SD of 6 rats in diabetes and 7 rats each in normal and diabetes + curcumin groups. *P < 0.05 compared to normal, and ^#^P < 0.05 compared to diabetes + curcumin.

The endothelial growth factor, VEGF, was elevated by over 2 fold in the retina obtained from diabetic rats (Figure 7). Supplementation with curcumin prevented diabetes-induced increase in VEGF; the values obtained from normal control and diabetes + curcumin rats were not significantly different from each other.

Similar beneficial effects of curcumin supplementation were obtained on diabetes-induced increased protein expression of retinal IL-1β, p65 subunit of NF-*k*B and VEGF (data not shown).

### Severity of hyperglycemia

The severity of hyperglycemia, as measured by body weight of the rats and their blood glucose and 24 hour urine volumes, was strikingly increased in the diabetes group compared with the normal control group. Curcumin did not ameliorate the severity of hyperglycemia in diabetic rats; the body weight, blood glucose and urine volumes were comparable between the two diabetic groups (diabetes and diabetes + curcumin), and these parameters were significantly different (P < 0.001) from the normal control group (Table 1). The average curcumin consumption in diabetic rats, calculated from the daily average food intake, was about 16 mg/day.

**Table 1:** Effect of curcumin on the severity of hyperglycemia in rats

	Body weight (g)	Food Consumption (g/day)	Blood glucose (mg/dl)	Urine volume (ml/24 hour)
Normal	255 ± 17	24.7 ± 6.0	111 ± 18	8 ± 3
Diabetes	216 ± 12*	36.6 ± 6.1*	469 ± 81*	53 ± 18*
Diabetes + Curcumin	200 ± 30^*#^	32.2 ± 3.2^*#^	451 ± 123^*#^	55 ± 14^*#^

Twenty four hour urine excretion (measured over 3 consecutive days) was quantified at 6 weeks of diabetes. Values are mean ± SD of 8 rats each in normal group and diabetes + curcumin group and 7 rats in diabetes group. *P < 0.02 compared to normal, ^#^P > 0.02 compared to diabetes.

## Discussion

This is the first report showing that curcumin, a polyphenol, has beneficial effects on retinal metabolic abnormalities, including oxidative stress and inflammation, which are considered to be important in the development of retinopathy in diabetes. These results raise the possibility that curcumin may help inhibit the development of diabetic retinopathy.

The antioxidant capacity, a measure of the total protective antioxidant mechanisms (both for preventing the production of free radicals and for repairing oxidative damage) [23], of curcumin has been considered to be mediated via its beneficial effects on the antioxidant defense system, the scavenging of free radicals and/or via preventing lipid peroxidation and it is at least 10 times more active as an antioxidant than vitamin E [24]. Diabetes decreases the total antioxidant capacity of the retina [21], and here we provide data showing that the administration of curcumin can prevent diabetes-induced decrease in the total antioxidant capacity of the retina. This suggests that curcumin has a potential to inhibit overall oxidative damage experienced by the retina in diabetes.

Administration of curcumin decreases diabetes-induced increase in retinal 8-OHdG levels. In support, recent studies by Farhangkhoee et al [25] have shown that curcumin treatment decreases diabetes-induced increased 8-OHdG immunoreactivity in the heart. 8-OHdG has been implicated in the pathogenesis of diabetic retinopathy; inhibition of increased retinal capillary cell apoptosis and the development of diabetic retinopathy by lipoic acid are considered to be mediated via inhibition of increased retinal 8-OHdG levels [25]. The role of increased retinal 8-OHdG levels in the pathogenesis of diabetic retinopathy is further strengthened by the studies showing that overexpression of mitochondrial superoxide dismutase (MnSOD) inhibits increase in retinal 8-OHdG levels [8,21]. Inhibition of diabetes-induced elevated retinal 8-OHdG levels by curcumin suggests that curcumin could inhibit the development of diabetic retinopathy, in part, via inhibiting accumulation of oxidized DNA in the retina.

GSH, an intracellular antioxidant, is important in antioxidant defense, nutrient metabolism, and regulation of cellular events, including gene expression, apoptosis and cytokine production [26]. Decreased GSH levels are observed in the retina in diabetes, and antioxidants and MnSOD overexpression inhibit such diabetes-induced decreases in retinal GSH [22,27]. Here, we provide data demonstrating that curcumin administration has partial beneficial effect on diabetes-induced decrease in retinal GSH; the GSH levels in curcumin-treated diabetic rats remained significantly lower than those in the normal control rats, but were significantly higher than diabetic rats. Others have shown that curcumin administration increases defense against oxidative stress in the liver via a GSH-linked mechanism [28]. Our failure to achieve complete restoration of retinal GSH levels could be that higher levels of curcumin may be essential to regulate retinal GSH metabolism in diabetes, and the rats in our experiment were not able to achieve such levels. Or, there could be compartmentalization of GSH in the retina, and levels could possibly be normalized in the cells associated with the development of diabetic retinopathy.

The therapies that inhibit diabetes-induced nitrotyrosine accumulation in the retina are shown to inhibit diabetic retinopathy [22,29,30]. Here we demonstrate that curcumin can prevent diabetes-induced increase in nitrotyrosine levels in the retina. In support, curcumin has been reported to inhibit diabetes-induced increased nitrotyrosine immunostaining in the heart [25], and also is postulated to exert its neuroprotective effects and prevent alcohol-induced liver damage via regulating peroxynitrite levels [31,32].

Diabetic retinopathy shares similarities with low level chronic inflammatory disease, and subclinical inflammation is considered to play a role in the vascular lesions characteristic of diabetic retinopathy [33]. The levels of cytokines, including IL-1β, IL-6 and IL-8, are increased in the vitreous of patients with proliferative diabetic retinopathy and in the retina from diabetic rats and mice [11,22,34]. The capillaries become nonperfused and ischemic and the number of platelet-fibrin thrombi increases; these pro-inflammatory changes and leukostasis constitute as some of the earliest changes observed in the retina of diabetic animals [10]. ROS are considered as strong stimuli for the release of cytokines, and IL-1β itself can trigger signaling cascades resulting in excessive ROS [14]. Our data demonstrate that curcumin, a compound with both anti-inflammatory and antioxidant properties, prevents diabetes-induced increase in IL-1β. This suggests that curcumin could inhibit the development of diabetic retinopathy by inhibiting both, proinflammatory cytokines and oxidative stress.

IL-1β induces the expression of various genes whose promoters are regulated through complex interactions with NF-*k*B [35]. NF-*k*B, a redox sensitive factor and a key regulator of antioxidant enzymes, can initiate transcription of many genes involved in apoptosis [36], and curcumin is a potent blocker of NF-*k*B activation [37]. We have shown that NF-*k*B is activated in the retina and its capillary cells in diabetes, and its activation is an early event in the development of retinopathy that is sustained when retinal capillary cell death is accelerating, and histopathology is developing [29]. In the pathogenesis of diabetic retinopathy activation of NF-*k*B is reported to trigger a developing pro-apoptotic program in retinal pericytes [38], and accelerated apoptosis can predict the development of retinopathy in diabetes [39]. Here we provide clear evidence that curcumin administration inhibits the activation of NF-*k*B, accumulation of 8-OHdG and nitrotyrosine in the retina in diabetes. This raises a possibility that curcumin can inhibit apoptosis of retinal capillary cells, a predictor of the development of diabetic retinopathy [39].

Diabetic retinopathy is accompanied by elevation in various angiogenic factors, and VEGF, a hypoxia-induced growth factor, is considered to play a pivotal role in the increased permeability and angiogenesis seen in diabetic retinopathy [15]. Oxidative stress is considered to regulate diabetes-induced retinal VEGF levels [40]. Our data clearly show that curcumin administration inhibits increased VEGF levels in the retina. In agreement, others have shown that curcumin can abolish IL-18 induced increase in VEGF production [41].

The beneficial effects of curcumin observed in the present study were achieved without amelioration of the severity of hyperglycemia in diabetic rats; the blood glucose, urine volume and body weights were similar in diabetic rats and diabetes + curcumin rats.

Curcumin is shown to be safely tolerated, clinical trials using up to 8000 mg curcumin per day for 3 months have shown no toxicity to curcumin [42]. Based on the food consumption, the rats in our experiment consumed about 16 mg curcumin per day (about 80 mg/kgBW), and this concentration is within the range of the concentration used for clinical trials. However, curcumin has poor oral bioavailability [43] that could limit its full potential in the retina. To increase its bioavailability, curcumin can be encapsulated in liposomes [44]; and that could allow the use of higher concentration to possibly inhibit diabetic retinopathy. Although, due to tissue limitations, direct measurements of curcumin levels in the retina were not made, the beneficial effects of curcumin observed in our study imply that curcumin was able to cross the blood-retina barrier. This is supported by others showing that curcumin can cross the blood-brain barrier in mice, and brain curcumin levels achieved are comparable to the levels achieved in the plasma [45].

## Conclusion

The pathogenesis of diabetic retinopathy is complex. Curcumin, a common food additive, has beneficial effects in experimental studies of the diseases that are characterized by increased oxidative stress and inflammatory reactions supporting its clinical use. Our studies are the first to show that curcumin can inhibit diabetes-induced retinal abnormalities that are postulated in the development of diabetic retinopathy. Thus, curcumin appears to be a useful adjunct therapy to possibly inhibit the development/progression of retinopathy, the sight threatening complication faced by diabetic patients.
